# Transcatheter versus Surgical Aortic Valve Replacement after Previous Cardiac Surgery: A Systematic Review and Meta-Analysis

**DOI:** 10.1155/2018/4615043

**Published:** 2018-04-05

**Authors:** Sharaf-Eldin Shehada, Yacine Elhmidi, Öznur Öztürk, Markus Kasel, Antonio H. Frangieh, Fanar Mourad, Jaroslav Benedik, Jaafar El Bahi, Mohamed El Gabry, Matthias Thielmann, Heinz Jakob, Daniel Wendt

**Affiliations:** ^1^Department of Thoracic and Cardiovascular Surgery, West-German Heart and Vascular Center Essen, University of Duisburg-Essen, Duisburg, Germany; ^2^Department of Cardiovascular Surgery, German Heart Center Munich, Technical University Munich, Munich, Germany; ^3^Department of Cardiovascular Surgery, Klinikum Bogenhausen, Munich, Germany; ^4^Department of Cardiology, German Heart Center Munich, Technical University Munich, Munich, Germany

## Abstract

**Aim:**

Aortic valve replacement (AVR) in patients with prior cardiac surgery might be challenging. Transcatheter aortic valve replacement (TAVR) offers a promising alternative in such patients. We therefore aimed at comparing the outcomes of patients with aortic valve diseases undergoing TAVR versus those undergoing surgical AVR (SAVR) after previous cardiac surgery.

**Methods and Results:**

MEDLINE, EMBASE, and the Cochrane Central Register were searched. Seven relevant studies were identified, published between 01/2011 and 12/2015, enrolling a total of 1148 patients with prior cardiac surgery (97.6% prior CABG): 49.2% underwent TAVR, whereas 50.8% underwent SAVR. Incidence of stroke (3.8 versus 7.9%, *p*=0.04) and major bleeding (8.3 versus 15.3%, *p*=0.04) was significantly lower in the TAVR group. Incidence of mild/severe paravalvular leakage (14.4/10.9 versus 0%, *p* < 0.0001) and pacemaker implantation (11.3 versus 3.9%, *p*=0.01) was significantly higher in the TAVR group. There were no significant differences in the incidence of acute kidney injury (9.7 versus 8.7%, *p*=0.99), major adverse cardiovascular events (8.7 versus 12.3%, *p*=0.21), 30-day mortality (5.1 versus 5.5%, *p*=0.7), or 1-year mortality (11.6 versus 11.8%, *p*=0.97) between the TAVR and SAVR group.

**Conclusions:**

TAVR as a redo procedure offers a safe alternative for patients presenting with aortic valve diseases after previous cardiac surgery especially those with prior CABG.

## 1. Introduction

Since decades, surgical aortic valve replacement (SAVR) has been considered as the gold standard for patients presenting with severe aortic stenosis (AS) [1]. SAVR can be performed either through conventional or minimal access methods [2, 3]. Patients with prior cardiac surgery and symptomatic aortic stenosis, especially those patients with previous coronary artery bypass grafting (CABG), were at higher risk. Transcatheter aortic valve replacement (TAVR) has been established as an equivalent alternative to surgical AVR in high-risk patients. Moreover, TAVR is currently evaluated even in intermediate-risk patients [4, 5]. Especially in the redo situation, TAVR decreases the risk of patent graft injury, which has been reported to be as high as 5% [6, 7]. While the use of TAVR is increasing worldwide, there is a current debate whether TAVR is superior to conventional SAVR in patients with previous cardiac surgery. Only few studies have been published comparing either the results of TAVR only [8–13] or SAVR only [14–17] in patients presenting with prior cardiac surgery. Some other studies matched and compared the results of both treatment modalities in redo patients [18–24]. Therefore, the purpose of this meta-analysis was to compare the outcomes in patients with prior cardiac surgery who underwent TAVR versus a conventional SAVR.

## 2. Methods

### 2.1. Data Collection and Inclusion Criteria

Based on the PRISMA guidelines [25], MEDLINE, EMBASE, and the Cochrane Central Register of Controlled Trials were searched from December 2015. Two investigators (Sharaf-Eldin Shehada and Yacine Elhmidi) independently assessed the relevant publications for eligibility through the title or the abstract of each publication. Only studies and articles using the following medical subject heading terms were evaluated: transcatheter aortic valve implantation/replacement, surgical/conventional aortic valve replacement, aortic valve stenosis, prior/previous cardiac surgery, previous coronary artery bypass grafting, and aortic valve replacement as a redo procedure after cardiac surgery. References of all relevant articles were also included in an additional search.

Inclusion criteria were as follows: (1) articles published between January 2011 and December 2015, (2) studies evaluating the impact of previous cardiac surgery especially those with a history of coronary artery bypass grafting in patients with severe aortic stenosis, (3) articles which compared TAVR and SAVR after prior CABG, (4) only studies including at least 40 patients in each group, (5) studies presenting VARC criteria, and (6) only articles written in English language.

### 2.2. Definition of Outcomes

Outcomes are defined based on the included studies, whereas only evaluated endpoints in the initial studies were collected and evaluated for this meta-analysis. The primary endpoints of our meta-analysis were as follows: (1) early (defined as inhospital or 30-day mortality based on the included studies), one-year mortality, and overall mortality (defined as all-cause mortality at the time of follow-up in each individual study, which varies between 6 and 48 months), (2) incidence of stroke, (3) acute kidney injury, and (4) major adverse cardiovascular events (MACE), according to VARC II [26]. Secondary endpoints included (1) incidence of major bleeding (including operative revision), (2) incidence of pacemaker implantation, (3) incidence of paravalvular leakage, (4) procedural times, and (5) the length of hospital stay.

### 2.3. Statistical Analysis

Continuous variables were expressed as mean ± standard deviation (SD) or median with interquartile range (IQR) (25–75th percentiles). Categorical variables were presented as numbers and percentages. The meta-analysis was performed using the Review Manager 5.3 software package (Nordic Cochrane Centre, Copenhagen, Denmark). Pooled estimation of odds ratios (ORs) with their 95% confidence intervals (CIs) was calculated using the Mantel–Haenszel method in cases of absence of heterogeneity between the compared studies. Heterogeneity of the studies was assessed with the *I*^2^ index, which indicates 25%, 50%, and 75% as low, moderate, and high heterogeneity, respectively. If significant heterogeneity between the studies was detected, the DerSimonian and Laird random-effect methods were used. Sensitivity analysis was performed by eliminating each study at a time to assess the influence of any included study on the results. All reported *P*-values are two-sided, and a value of *P* < 0.05 was considered statistically significant.

## 3. Results

**Figure 1:**
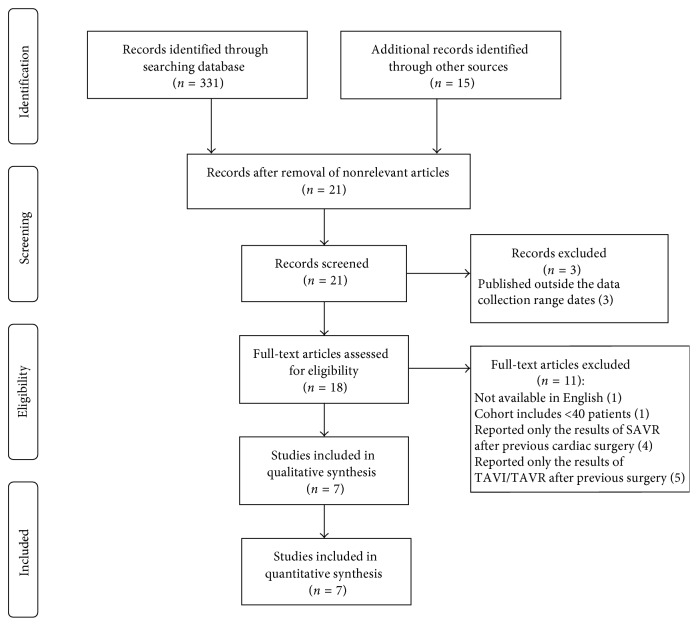
Study flowchart.

The primary search revealed 346 potential relevant studies and articles. After removal of nonrelevant articles, a total of 21 studies remained. Three studies were excluded, as they did not fulfill the inclusion criteria (published after December 2015). Hence, 18 studies were evaluated. After final exclusion, seven studies remained for the systematic meta-analysis (Figure 1). These seven studies compromised a total of 1148 patients with a history of previous cardiac surgery (coronary artery bypass grafting in 1121 (97.6%) patients), of whom 565 (49.2%) underwent TAVR and 583 (50.8%) underwent SAVR.  Table 1 summarizes the total incidences of the endpoints of the meta-analysis, and all baseline demographics and echocardiographic data of the included studies were summarized in Table 2. Patients enrolled were mainly males and nearly a half had diabetes. Overall, about 50% of patients presented with peripheral vascular disease. Patients' age ranged from 78.1 ± 5 to 82 ± 5.8 years in the TAVR cohort versus 70.6 ± 8 to 82.3 ± 6.2 years in the SAVR cohort. The STS PROM and logistic EuroSCORE ranged from 7.3 ± 2.7% to 24 ± 6% and 11.1 ± 2.8% to 36.4 ± 17.4% in the TAVR cohort versus 6.3 ± 6% to 19 ± 6% and 10.4 ± 3% to 33.8 ± 15.3% in the SAVR cohort, respectively.

**Figure 2:**
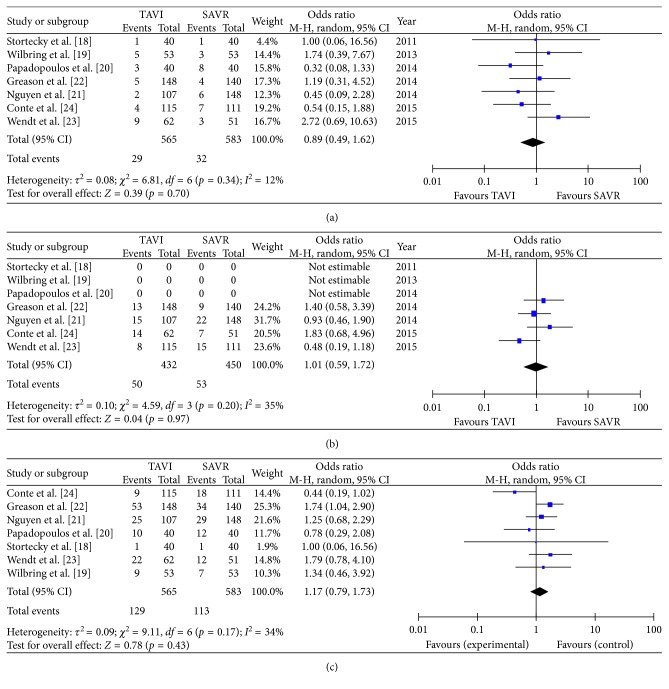
Meta-analytic comparison showing (a) early mortality rate between the TAVR and SAVR group, (b) one-year mortality between the TAVR and SAVR group, and (c) overall mortality between the TAVR and SAVR group.

**Figure 3:**
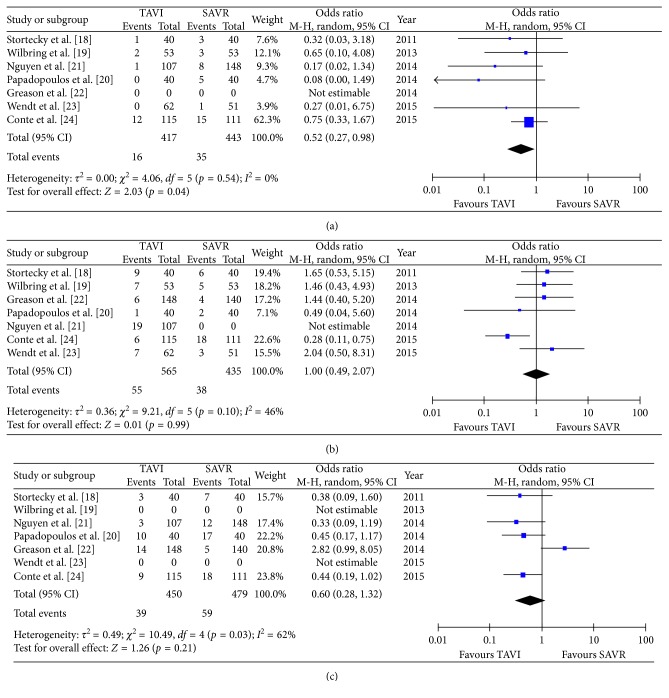
Meta-analytic comparison showing (a) incidence of stroke between the TAVR and SAVR group, (b) incidence of acute kidney injury between the TAVR and SAVR group, and (c) incidence of major adverse cardiovascular events between the TAVR and SAVR group.

There was no difference in early mortality (5.1% in TAVR versus 5.5% in SAVR patients: OR 0.89 (95% CI 0.49 to 1.62); *p*_heterogeneity_ = 0.7; *I*^2^ = 12%), without significant heterogeneity among the studies (Figure 2(a)); one-year mortality (11.6% versus 11.8%, OR 1.01 (95% CI 0.59 to 1.72); *p*_heterogeneity_ = 0.97; *I*^2^ = 35%), without significant heterogeneity among the studies (Figure 2(b)); and overall mortality (22.8% versus 19.4%, OR 1.17 (95% CI 0.79 to 1.73); *p*_heterogeneity_ = 0.43; *I*^2^ = 34%), without significant heterogeneity among the studies (Figure 2(c)), respectively. Interestingly, the incidence of stroke was significantly lower in the TAVR group (3.8%) compared to the SAVR group (7.9%, OR 0.52 (95% CI 0.27 to 0.98); *p*_heterogeneity_ = 0.04; *I*^2^ = 0%), without any significant heterogeneity among the evaluated studies (Figure 3(a)). Moreover, both groups did not differ in regard to acute kidney injury (9.7% versus 8.7%, OR 1.00 (95% CI 0.49 to 2.07); *p*_heterogeneity_ = 0.99; *I*^2^ = 46%) (Figure 3(b)) or major adverse cardiovascular events (8.7% versus 12.3%, OR 0.60, (95% CI 0.28 to 1.32); *p*_heterogeneity_ = 0.21; *I*^2^ = 62%) (Figure 3(c)).

**Figure 4:**
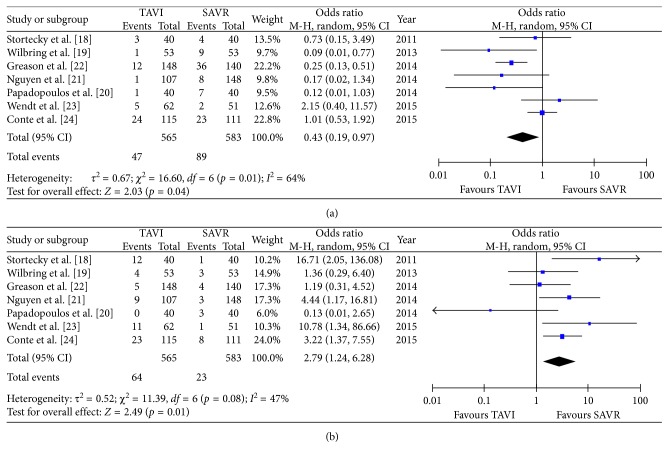
Meta-analytic comparison showing (a) incidence of major bleeding between the TAVR and SAVR group and (b) incidence of pacemaker implantation between the TAVR and SAVR group.

**Figure 5:**
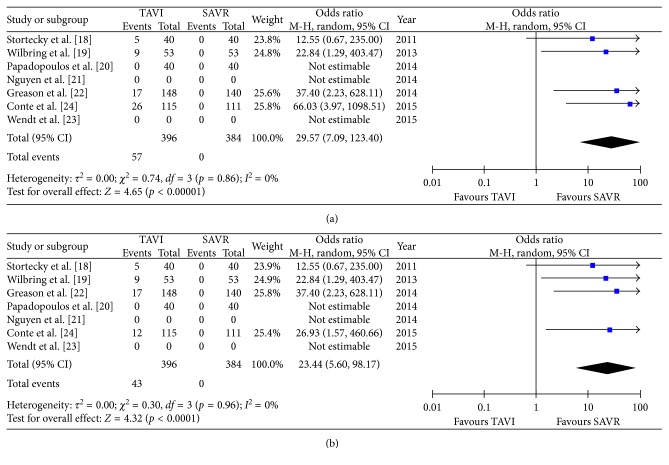
Meta-analytic comparison showing (a) incidence of mild-to-moderate paravalvular leakage between the TAVR and SAVR group and (b) incidence of moderate-to-severe paravalvular leakage between the TAVR and SAVR group.

**Figure 6:**
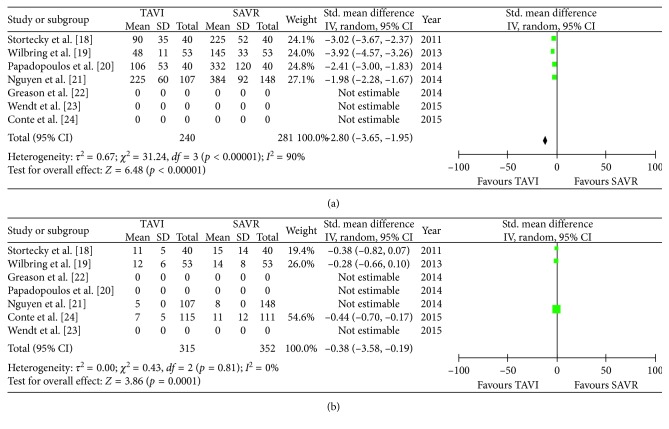
Meta-analytic comparison showing (a) procedural times in the TAVR and SAVR group and (b) length of hospital stays in the TAVR and SAVR group.

All secondary endpoints showed significant differences between both groups: the incidence of major bleeding was significantly lower in the TAVR group (8.3%) compared to the SAVR group (15.3%, OR 0.43 (95% CI 0.19 to 0.97); *p*_heterogeneity_ = 0.04; *I*^2^ = 64%); however, there was a significant heterogeneity among the evaluated studies (Figure 4(a)). Conversely, the incidence of permanent pacemaker implantation was significantly higher in the TAVR group (11.3% versus 3.9% patients, OR 2.79 (95% CI 1.24 to 6.28); *p*_heterogeneity_ = 0.01; *I*^2^ = 47%) (Figure 4(b)). Moreover, the incidence of both mild-to-moderate and moderate-to-severe paravalvular leakage was significantly higher in the TAVR group (Figures 5(a) and 4(b)) with no PVL reported in the SAVR group. Procedural time (OR 2.80 (95% CI 3.65 to 1.95); *p*_heterogeneity_ < 0.0001; *I*^2^ = 90%) (Figure 6(a)) and hospital stay (OR 0.38 (95% CI 0.58 to 0.19); *p*_heterogeneity_ < 0.0001; *I*^2^ = 0%) (Figure 6(b)) were significantly lower in the TAVR group.

## 4. Discussion

The current meta-analysis evaluates for the first time the outcomes of patients undergoing TAVR versus SAVR after a previous cardiac surgery. The main findings of this study were as follows: (1) there were no significant differences in early, one-year, or overall mortality between both groups. (2) Interestingly, SAVR patients were more likely to experience postoperative stroke compared to TAVR patients. (3) There was no difference in postoperative acute kidney injury between both groups. (4) TAVR patients experienced significantly higher rates of pacemaker implantation and paravalvular leakage.

Before the TAVR era, surgical aortic valve replacement as a redo procedure in patients with previous CABG has been considered as the gold standard therapy for patients presenting with symptomatic aortic stenosis. The procedure, however, could be challenging due to patent bypass grafts. Mortality has been reported up to 20% in high-risk patients [27, 28]. Although even lower mortality rates have been described [29], redo surgery is sometimes technically challenging due to severe adhesions with the risk of injury of the right ventricle, or patent graft injury, or the difficulty to achieve optimal myocardial protection even with the use of retrograde cardioplegia. Transcatheter aortic valve replacement has been established as an alternative therapy in patients with severe aortic stenosis, who were deemed to be at prohibitive risk for open-heart surgery. Moreover, TAVR presented promising results with lower intraprocedural complications and promising follow-up results in high-risk or even intermediate-risk patients [30].

Over the last years, there has been an ongoing debate about the advantages and disadvantages of TAVR over SAVR in primary aortic stenosis. The present study, however, aimed at evaluating the outcomes in a selected group of patients with previous cardiac surgery. Patients with prior cardiac surgery, by nature, show a higher risk, which is mainly reflected by the preoperative calculated risk scores. Interestingly, the current meta-analysis demonstrated a higher stroke rate in patients undergoing SAVR after prior cardiac surgery. Comparing those findings with previous reports, stroke rates vary between 5.7% [6] and 8% in the RECORD multicenter study [7]. Stroke might be caused by aortic cross clamping or calcium removal during surgical AVR, whereas in TAVR, the calcified aortic valve is pressed into the aortic wall, which could also cause stroke by calcified debris. Within the present meta-analysis, we did not observe any differences in the incidence of MACEs in both groups. There was no significant difference in the occurrence of acute kidney injury (AKI) rates in both groups, despite the use of contrast media in TAVR patients. In regard to acute kidney injury, previously published data demonstrated that, preoperative creatinine, the presence of peripheral vascular diseases, and blood transfusion are predictors for AKI after TAVR [31, 32]. The contrast media was, however, not a predictor for AKI.

In regard to the secondary outcomes of the present meta-analysis, the redo SAVR group experienced more major bleeding (15.3%) compared to TAVR patients (8.3%). Those results are in accordance with the previously reported one that evaluated the risk of reexploration for bleeding in case of redo surgery due to dissection leaving a row area and/or injury of the heart or grafts due to severe adhesions. This increased risk of major bleeding events in the redo situation has been shown to be a predictor of 30-day mortality in the multivariable analysis by Vohra et al. [6]. Patients undergoing TAVR experienced more mild-to-moderate and moderate-to-severe PVL compared to SAVR. This has been also shown in the PARTNER trial, which additionally demonstrated that moderate-to-severe PVL was associated with higher 1-year mortality (cardiac and noncardiac) and rehospitalization after TAVR [33]. Even in patients with mild PVL, the mortality rate was higher than in those with no PVL [33] with a clear advantage for SAVR. Having said that, TAVR with third-generation devices promises less PVL [34].

### 4.1. TAVR versus SAVR as a Redo Procedure

Currently, with improving devices, techniques, and encouraging recent results from TAVR in intermediate-risk patients [4, 5], the worldwide adoption of TAVR is becoming an important tool in the treatment of severe AS. However, patients with patent grafts presenting only with intermediate-risk scores are by nature a “higher risk” group due to possible harming of those patent grafts during redo surgery. Of note, patients undergoing TAVR or SAVR after previous CABG exhibited different mortality rates as calculated in the preoperative STS PROM or EuroSCORE. Moreover, previous reports discussed this important point and debate the role of STS or EuroSCORE in the decision-making between SAVR and TAVR. A previous study from Khaladj et al. evaluated the results of 349 patients who underwent SAVR after a history of CABG [16]. They reported that the early (inhospital or 30-day) mortality was not higher than 5% compared to the calculated STS and logistic EuroSCORE of 10 ± 4% and 32 ± 21%, respectively. Therefore, although all current risk-scoring systems have been updated recently, both the STS PROM and EuroSCORE overestimated the risk of mortality in those patients [35, 36]. The authors concluded that SAVR as a redo procedure after CABG can be performed with a lower mortality rate as predicted by STS or EuroSCORE [16].

In addition, those previous results were consistent with the results of the RECORD multicenter registry [7]. The investigators observed a lower early mortality of 4.4% in 113 patients who underwent an isolated SAVR after a history of CABG [7]. The authors concluded that a history of CABG should not be an indication for TAVR [7], although patients with prior CABG and especially those with patent grafts have an increased risk of graft injury. Interestingly, the present meta-analysis reported that TAVR patients experienced fewer strokes than SAVR patients in redo procedures. The decision, however, to choose either the TAVR or SAVR procedure in patients with prior surgery should be discussed in a “heart-team” and should include several factors including demographics, anatomical challenges, the presence of porcelain aorta, the number of patent grafts and, most importantly, the physical condition of the patient and, moreover, the individual patients' wish.

### 4.2. In Summary

The present meta-analysis showed no significant differences in early, one-year mortality, and overall mortality between TAVR and SAVR patients presenting with prior CABG surgery. SAVR patients demonstrated a lower rate of pacemaker and less mild-to-moderate PVL in comparison to TAVR patients in the redo situation. However, there was a higher rate of postprocedural stroke and bleeding in patients who underwent SAVR. TAVR offers an attractive, fast, and as safe alternative as SAVR for patients presenting with aortic stenosis after previous cardiac surgery, but the history of CABG per se should not be the only leading factor to decide for TAVR.

### 4.3. Study Limitations

The baseline characteristics were not similar in all included studies, and access site used for TAVR (e.g., transfemoral, transapical, transaortic, or trans-subclavian access) was not mentioned in all studies. The evaluated endpoints depend mainly on the presence or absence of each event in the included studies; for example, early mortality is evaluated as inhospital mortality in some studies and as 30-day mortality in other studies; moreover, overall mortality was mentioned in the studies at different follow-up times which varies between 6 and 48 months, that is why it should not be considered as an accurate result in this meta-analysis. The type of cardioplegia used in SAVR was also not mentioned in all the included studies. In addition, all evaluated articles did not present the rate of potential graft injury during redo surgery and if a patent LIMA graft was clamped during redo surgery for aortic stenosis. Finally, the included studies did not present the cause and site of bleeding (e.g., graft injury or right ventricular injury).

## Figures and Tables

**Table 1:** Summery of the total incidences of the endpoints of the meta-analysis.

	TAVR	SAVR	OR (95% CI)	*p* value
*Primary endpoints*				
Early mortality, *n* (%)	29/565 (5.1)	32/583 (5.5)	0.89 (0.49, 1.62)	*p*=0.7
One-year mortality, *n* (%)	50/432 (11.6)	53/450 (11.8)	1.01 (0.59, 1.72)	*p*=0.97
Overall mortality, *n* (%)	129/565 (22.8)	113/583 (19.4)	1.17 (0.79, 1.73)	*p*=0.43
Stroke, *n* (%)	16/417 (3.8)	35/443 (7.9)	0.52 (0.27, 0.98)	*p*=0.04
Acute kidney injury, *n* (%)	55/565 (9.7)	38/435 (8.7)	1.00 (0.49, 2.07)	*p*=0.99
Major adverse events, *n* (%)	39/450 (8.7)	59/479 (12.3)	0.60 (0.28, 1.32)	*p*=0.21
*Secondary endpoints*				
Major bleeding, *n* (%)	47/565 (8.3)	89/583 (15.3)	0.43 (0.19, 0.79)	*p*=0.04
Permanent pacemaker, *n* (%)	64/565 (11.3)	23/583 (3.9)	2.79 (1.24, 6.28)	*p*=0.01
Moderate paravalvular leakage, n (%)	57/396 (14.4)	0/384 (0)	29.57 (7.09, 123.4)	*p* < 0.0001
Severe paravalvular leakage, *n* (%)	43/396 (10.9)	0/384 (0)	23.44 (5.60, 98.17)	*p* < 0.0001
Procedural time (range), mean ± SD	(48 ± 11) to (225 ± 60)	(145 ± 33) to (384 ± 92)	−2.80 (−3.65, −1.95)	*p* < 0.0001
Hospital stay (range), mean ± SD	(5 ± 0) to (12 ± 6)	(8 ± 0) to (15 ± 14)	−0.38 (−0.58, −0.19)	*p* < 0.0001

**Table 2:** Baseline and echocardiography data of the included studies.

Year of publication	Stortecky et al. [18]		Wilbring et al. [19]		Papadopoulos et al. [20]		Nguyen et al. [21]		Greason et al. [22]		Wendt et al. [23]		Conte et al. [24]	
	2011		2013		2014		2014		2014		2015		2015	
Number of patients	80		106		80		255		288		113		226	
	TAVR	SAVR	TAVR	SAVR	TAVR	SAVR	TAVR	SAVR	TAVR	SAVR	TAVR	SAVR	TAVR	SAVR
Patients who underwent TAVR/SAVR	40	40	53	53	40	40	107	148	148	140	62	51	115	111
Age (mean ± SD)	78.2 ± 6	70.6 ± 8	78.1 ± 5.5	77.6 ± 2.7	81 ± 4	80 ± 3	79.8 ± 7.9	72.5 ± 8.8	80.7 ± 7	82.3 ± 6.2	78.7 ± 5.9	71.1 ± 10.8	82 ± 5.8	81 ± 5.9
Body mass index (mean ± SD)	27.4 ± 5	28 ± 5	27.9 ± 4.0	27.3 ± 4.2	N/A	N/A	27.2 ± 5	28.4 ± 5.3	N/A	N/A	27.1 ± 4.1	26.6 ± 3.7	N/A	N/A
Male (%)	32 (80)	33 (83)	26 (65)	35 (66)	29 (73)	29 (73)	81 (75.7)	116 (78.4)	120 (81)	111 (79)	43 (69.4)	38 (74.5)	91 (79.1)	87 (78.4)
Diabetes mellitus (%)	19 (48)	13 (33)	28 (52.8)	23 (43.4)	17 (42)	14 (35)	48 (44.9)	71 (48)	74 (50)	70 (50)	24 (38.7)	22 (43.1)	49 (42.6)	58 (52.3)
Systemic hypertension (%)	34 (85)	36 (90)	N/A	N/A	16 (40)	18 (45)	105 (98.1)	138 (93.2)	N/A	N/A	57 (91.9)	45 (88.2)	N/A	N/A
NYHA III/IV (%)	26 (65)	26 (65)	N/A	N/A	N/A	N/A	N/A	N/A	140 (94.6)	131 (93.6)	N/A	N/A	96 (83.5)	98 (88.3)
Coronary artery disease (%)	40 (100)	40 (100)	53 (100)	53 (100)	33 (83)	30 (75)	86 (80.4)	126 (85.1)	148 (100)	140 (100)	N/A	N/A	115 (100)	111 (100)
Myocardial infarction (%)	20 (50)	14 (35)	N/A	N/A	N/A	N/A	N/A	N/A	60 (41)	62 (45)	N/A	N/A	N/A	N/A
PCI (%)	N/A	N/A	N/A	N/A	N/A	N/A	N/A	N/A	59 (40.4)	50 (35.7)	N/A	N/A	49 (42.6)	50 (45)
Renal failure/dialysis (%)	N/A	N/A	35 (66)	32 (60.4)	20 (50)	16 (40)	N/A	N/A	N/A	N/A	12 (23.5)	13 (21)	10 (8.7)	8 (7.3)
Cerebrovascular disease (%)	4 (10)	6 (15)	10 (18.9)	8 (15.1)	9 (23)	8 (20)	41 (38.3)	45 (30.4)	49 (35.5)	40 (29.4)	N/A	N/A	37 (32.7)	32 (28.8)
Peripheral vascular disease (%)	21 (53)	9 (23)	N/A	N/A	13 (33)	11 (27)	48 (44.9)	42 (28.4)	75 (50.7)	67 (48.6)	32 (52)	15 (29.4)	47 (41.6)	57 (51.8)
COPD (%)	7 (17.5)	7 (17.5)	5 (9.4)	4 (7.5)	9 (23)	8 (20)	55 (51.4)	44 (29.7)	67 (45.3)	58 (41.4)	16 (25)	17 (33.3)	48 (41.7)	43 (38.7)
STS score (median or mean ± SD)	7.6 ± 7	6.3 ± 6	N/A	N/A	24 ± 6	19 ± 6	11.8	7.1	11.8 ± 3.3	12 ± 3.1	12 ± 10	7.1 ± 5.2	7.3 ± 2.7	8.0 ± 3.5
EuroSCORE (mean ± SD)	33.5 ± 17	20.2 ± 14	29.9 ± 14	26.4 ± 12.9	11.1 ± 2.8	10.4 ± 3	N/A	N/A	34.6 ± 16.8	33.8 ± 15.3	36.4 ± 17.4	22.2 ± 17.5	25.6 ± 16.2	24.2 ± 15.8
Previous CABG (%)	40 (100)	40 (100)	48 (90.6)	49 (92.4)	N/A	N/A	107 (100)	148 (100)	148 (100)	140 (100)	59 (95.2)	36 (70.6)	115 (100)	111 (100)
Previous AVR (%)	0	0	5 (9.4)	4 (7.6)	N/A	N/A	0	0	0	0	2 (3.2)	10 (19.6)	0	0
Other cardiac surgery (%)	0	0	0	0	N/A	N/A	0	0	0	0	1 (1.6)	5 (9.8)	0	0
Mean AVA (cm^2^) (mean ± SD)	N/A	N/A	N/A	N/A	0.63 ± 0.29	0.68 ± 0.31	0.7 ± 0.12	0.76 ± 0.23	0.68 ± 0.2	0.66 ± 0.2	N/A	N/A	N/A	N/A
MAVPG (mmHg) (mean ± SD)	39 ± 15	48.6 ± 16	44 ± 4	55 ± 10	57 ± 21	51 ± 16	43.3 ± 13.0	42.0 ± 12.6	65.9 ± 20.3	68.9 ± 22.2	N/A	N/A	N/A	N/A
LVEF % (mean ± SD)	46.5 ± 15	49.8 ± 14	N/A	N/A	48 ± 14	47 ± 12	43.3 ± 13.1	46.9 ± 14.8	50.4 ± 13.3	52.2 ± 11.5	48.1 ± 13	49.9 ± 12.3	N/A	N/A

TAVR/SAVR, transcatheter/surgical aortic valve replacement; NYHA, New York Heart Association; PCI, percutaneous coronary intervention; COPD, chronic obstructive pulmonary disease; CABG, coronary artery bypass grafting; AVA, aortic valve area; MAVPG, mean aortic valve pressure gradient.

## Abbreviations

AKI: Acute kidney injury
AS: Aortic stenosis
AVR: Aortic valve replacement
CABG: Coronary artery bypass grafting
CI: Confidence interval
EuroSCORE: European System for Cardiac Operative Risk Evaluation
LVEF: Left ventricular ejection fraction
MACE: Major adverse cardiovascular events
OR: Odds ratio
PVL: Paravalvular leakage
TAVR: Transcatheter aortic valve replacement
SAVR: Surgical aortic valve replacement
STS PROM: Society of Thoracic Surgery
VARC: Valve Academic Research Consortium.
